# Effect of Cryogenic Grinding on Fatigue Life of Additively Manufactured Maraging Steel

**DOI:** 10.3390/ma14051245

**Published:** 2021-03-05

**Authors:** Arunachalam S. S. Balan, Kannan Chidambaram, Arun V. Kumar, Hariharan Krishnaswamy, Danil Yurievich Pimenov, Khaled Giasin, Krzysztof Nadolny

**Affiliations:** 1 Department of Mechanical Engineering, National Institute of Technology Karnataka, Mangaluru 575025, India; balan@nitk.edu.in; 2 Department of Automotive Engineering, School of Mechanical Engineering, Vellore Institute of Technology, Vellore 632014, India; 3 Department of Manufacturing Engineering, School of Mechanical Engineering, Vellore Institute of Technology, Vellore 632014, India; npkanna@yahoo.com; 4 Department of Mechanical Engineering, Indian Institute of Technology Madras, Chennai 600025, India; hariharan@iitm.ac.in; 5 Department of Automated Mechanical Engineering, South Ural State University, Lenin Prosp.76, 454080 Chelyabinsk, Russia; danil_u@rambler.ru; 6 School of Mechanical and Design Engineering, University of Portsmouth, Portsmouth PO1 3DJ, UK; Khaled.giasin@port.ac.uk; 7 Department of Production Engineering, Faculty of Mechanical Engineering, Koszalin University of Technology, Raclawicka 15-17, 75-620 Koszalin, Poland; krzysztof.nadolny@tu.koszalin.pl

**Keywords:** maraging steel, additive manufacturing, cryogenic grinding, residual stress, surface roughness, fatigue, microhardness

## Abstract

Additive manufacturing (AM) is replacing conventional manufacturing techniques due to its ability to manufacture complex structures with near-net shape and reduced material wastage. However, the poor surface integrity of the AM parts deteriorates the service life of the components. The AM parts should be subjected to post-processing treatment for improving surface integrity and fatigue life. In this research, maraging steel is printed using direct metal laser sintering (DMLS) process and the influence of grinding on the fatigue life of this additively manufactured material was investigated. For this purpose, the grinding experiments were performed under two different grinding environments such as dry and cryogenic conditions using a cubic boron nitride (CBN) grinding wheel. The results revealed that surface roughness could be reduced by about 87% under cryogenic condition over dry grinding. The fatigue tests carried out on the additive manufactured materials exposed a substantial increase of about 170% in their fatigue life when subjected to cryogenic grinding.

## 1. Introduction

Maraging steel falls under a superior class of steels with high strength and toughness. Typically, they have a high amount of nickel along with other alloying elements such as cobalt, molybdenum and a small amount of titanium and carbon. As the high strength and fracture toughness of this material can be retained over a range of temperatures, they are regularly being utilized in aviation, maritime vessels and tooling ventures [1]. The high strength of maraging steel is normally associated with the precipitation of intermetallic compounds. During the heat treatment, elements like Ni, Ti, and Mo, which are dissolved in the matrix segregate out and form compounds of Ni_3_T, Ni_3_Mo, Fe_2_Mo, and Fe_7_Mo_6_ and thus aids precipitation hardening.

At present, the manufacturing industries are looking into the prospects of additive techniques due to their high efficiencies and lower environmental impacts [2]. Besides, the additive manufactured aluminium alloys and composites are found to be exhibiting better mechanical properties [3]. In general, additive manufacturing is associated with high heat input and a rapid cooling rate. This results in complex residual stresses and heterogeneous microstructure. Post-heat treatment is usually employed to relieve residual stresses. The rapid cooling rate followed by heat treatment is similar to rapid quenching followed by aging adopted to manufacture maraging steels. Therefore, maraging steel is one of the ideal materials to be produced additively using high heat input direct deposition process. Commercially, the electro-optical system (EOS) has developed alloy powder (commercial name-MS1) whose composition is equivalent to that maraging steel. Maraging steel can be subjected to aging [4,5] post additive manufacturing for precipitate hardening and improvement of mechanical properties. Feuerhahn et al. [6] reported that aging helps in attaining uniform microstructure, improved hardness and reducing crack propagation rate. However, the aging parameters post quenching in the conventional casting route cannot be directly applied to the AM process as the microstructure and internal residual stress state of the alloy are different in both routes. When the maraging steel was subjected to aging between 610° and 690 °C, the reversion of martensite to austenite phase occurs [7,8]. The surface roughness generated on the additive manufactured component is found to be one of the major drawbacks. Hashimoto et al. [9] discussed the influence of surface property on product durability. The microcracks formed with high surface roughness act as stress concentration zones that accelerate the crack growth rate under structural load, especially fatigue [10,11].

Fatigue behaviour in AM parts is further deteriorated due to complex residual stresses formed as a result of a strong thermal gradient between layers [12]. Tensile residual stress (TRS) remained to be maximum on the uppermost layer and decreases with depth. It was also noted that there was a sudden transformation of stress from tensile to compressive which will affect the functionality of the parts manufactured [13,14]. In general, post-heat treatment is often employed to relieve residual stress. In the case of maraging steel, the aging treatment to facilitate intermetallic precipitates can greatly reduce the residual stress. Therefore, the present study aims to alleviate the other issue of improving the surface property of the component.

The surface roughness is playing a dominant role when compared to build orientation and internal defects of AM parts in predicting their fatigue life [15]. An investigation carried out to study the effect of vibratory grinding on the surface roughness and fatigue life of Ti-6Al-4V AM parts revealed that about 95% reduction in surface roughness and a considerable improvement in the fatigue life [16]. The comparison of different post-processing technologies for selective laser melting (SLM) generated 316L steel parts were executed and about 95% reduction in surface roughness was reported for the grinding [17].

Fatigue life is dependent upon the residual stresses, internal defects and surface roughness generated during the manufacturing process. Under typical cyclic load, tensile residual stress is detrimental. For instance, Kawagoishi et al. [18] studied the effect of shot peening on the fatigue life of aluminium alloys and maraging steel, the compressive residual stress induced in the material as a result of shot peening was found to improve the fatigue life. The shot peening, in addition to compressive residual stress, would introduce surface irregularities too. Other known processes such as grinding can be employed to improve the surface quality and reduce the residual stresses in AM parts. The influence of grinding parameters on the surface quality of the material was exhaustively investigated and reported by researchers [19,20]. The grinding tool/workpiece interface experiences very high temperature, which when can lead to the formation of untampered martensite on cooling. Such re-hardening burn is undesirable, especially in maraging steels which is sensitive to post-heat treatment. The lubrication plays an important role in the grinding process [21]. The effect of minimum quantity lubrication on grinding was investigated and it was found that coolant flow rate and delivery pressure has got a greater impact on determining the grinding performance [22,23,24]. The machining experiments under a cryogenic environment were found to render better surface integrity than any other form of the cooling technique [25]. Cryogenic grinding was able to produce a quality product with less material and also reduced the grinding force when compared to dry and wet grinding [26]. It has been shown that cryogenic grinding is efficient in eliminating tensile residual stress in the surface by reducing the cutting zone temperature and surface roughness [27,28,29]. Consequently, fatigue life improves, as demonstrated by Fredj and Sidhom [30] in AISI 304 stainless steel.

In this study, the grinding experiments were performed on additively manufactured maraging steel with different levels of depth of cut and speed under two different (dry, cryogenic) environments. An optimum grinding condition was determined. The additively manufactured maraging steel was ground with optimum parameters and subjected to a fatigue test to evaluate the influence of the grinding environment. Thus, the research objectives of the present study are to (a) analyse the effect of aging on tensile properties of DMLS maraging steel (b) investigate the effect of grinding environment on the forces generated, temperature, surface roughness, microhardness and residual stress induced by performing experiments under dry and cryogenic environment (c) develop regression models and (d) optimization of grinding parameters such as speed, depth of cut and environment for favourable results.

## 2. Materials and Methods

The specimens were printed with MS1 powder feedstock on EOSINT M280_400W (EOS GmbH Electro Optical Systems, Krailling, Germany) equipped with an Ytterbium fibre laser (TOPTICA Photonics AG, Graefelfing, Germany). A laser intensity of 400 W with a scanning speed of 7000 mm/s was adopted and all specimens were fabricated with a layer thickness of 40 μm. The composition of MS1 powder provided by the supplier is presented in Table 1. A total of 20 samples, oriented at 0° between the build stand and a longitudinal axis were produced.

**Figure 1:**
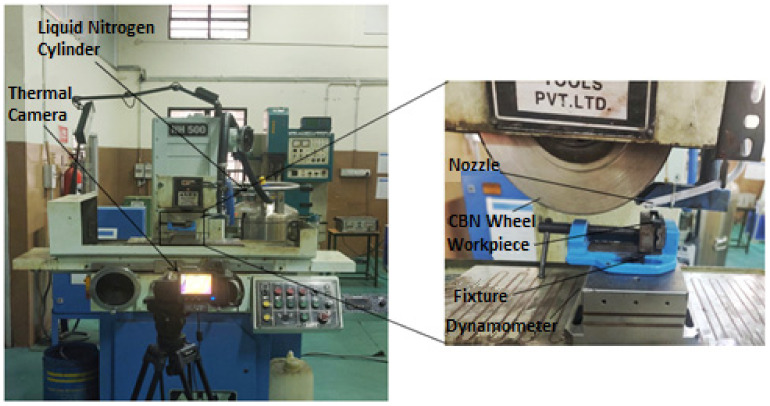
Experimental setup.

Literature revealed that aging at 520 °C for 8 h had given a better result in terms of strength. Hence, all specimens were solution annealed at 820 °C and later aged 520 °C for 8 h, which forms a densely populated dispersion of dislocation hindering precipitates, thereby increasing the strength and hardness of the material. The maraging steel samples were cut into small pieces (around 18 in number) for parametric experimental studies. A surface grinder, NH500 with magnetic chuck (Alex M/c Tools, Mumbai, India), as shown in Figure 1 was utilized for the grinding operation. CBN grinding wheel (Grindtec India, Gujrat, India) of diameter 200 mm and thickness 10 mm was used. The nozzle from the liquid nitrogen tank was attached near the grinding wheel in such a way that it falls directly on the tool-workpiece interface, as shown in Figure 1. Liquid nitrogen stored at 1 bar pressure was delivered at a flow rate of 15 L/min. The grinding parameters are specified in Table 2. For force measurement, the Kistler dynamometer (Kistler, Winterthur, Switzerland) was attached to a magnetic chuck over which samples were mounted of the fixture. FLIR T420 Thermal camera (TEquipment, NJ, USA) is used to measure the heat generated in different conditions. The surface roughness of the printed samples and grounded samples was measured using the Marsurf GD120 (Mahr, Gottingen, Germany) surface profilometer. A sample length of 5.6 mm was considered, the stylus was driven at the speed of 0.5 mm/rev. The surface roughness was measured at five different places and the average of those observations was presented in this study. The induced residual stress was measured using a μ-X360s portable X-ray residual stress analyser (Pulstec, Hamamatsu, Japan); the residual stresses are estimated using the “cos α” method with accuracy in the range of ±2 MPa.

Tensile and fatigue testing was performed according to ASTM standards E8 [31] and E466 [32] using INSTRON 8801 servo-hydraulic machine (INSTRON, Norwood, MA, USA). For both dry and cryogenic condition, three sets of samples were tested. Tensile tests were conducted at a speed of 2 mm/min for all samples. For better-quality gripping, knurling was done on the grip section of the fatigue specimen. Fatigue testing was done in a fully reversed condition (R = −1) with a frequency of 5 Hz. The maximum and minimum stress for the fatigue test was calculated from the tensile data and a magnitude of 675 MPa is applied during the fatigue testing. Upon failure, the fractured surfaces were taken for the fractography analysis. FE-SEM QUANTA FEG250 (Thermo Fisher Scientific, New York, NY, USA) was utilized for this purpose.

## 3. Results and Discussion

### 3.1. Grinding Forces

**Figure 2:**
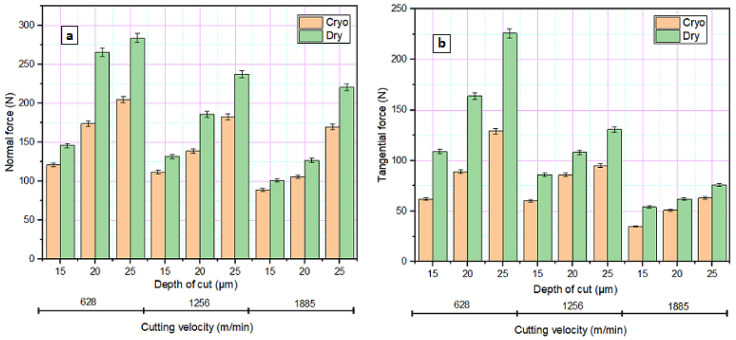
Variation in grinding force under different grinding environments. (**a**) Normal force and (**b**) Tangential force.

The impact of two different grinding conditions on the normal (F_z_) and tangential (F_x_) component of grinding force are shown in Figure 2. A substantial reduction in both components of the grinding force is observed, as the velocity of the wheel increases from 628 to 1885 m/min. At higher velocities, material removal occurs through steady-state mode rather than the ploughing mode, which reduces the grinding force. As the grinding depth increases, both F_z_ and F_x_ increase independent of velocity. At higher velocity and depth of cut, the thickness of the undeformed chip rises, thereby increasing the grinding force [29]. In addition to undeformed chip thickness, a larger number of arbitrarily oriented abrasive particles will establish contact with the workpiece as the depth of cut increases. The associated increase in grit depth of cut enhances shearing and ploughing action on the surface which further contributes to increased grinding force [33,34]. The temperature developed in the grinding area plays an essential role in governing the grinding force since the sharpness of the grit depends on the temperature and adhesion of the material [26].

It is obvious that the reduced temperatures obtained in cryogenic grinding considerably reducing the grinding forces when compared to the dry environment. This low temperature achieved by cryogenic grinding and continued possession of the abrasive grit sharpness due to reduced wear and plastic distortion contributed to reduced grinding force [35]. During cryogenic grinding, the low temperature favoured material removal through micro fracturing rather than shearing and ploughing thereby decreasing the wheel load [36].

### 3.2. Temperature

**Figure 3:**
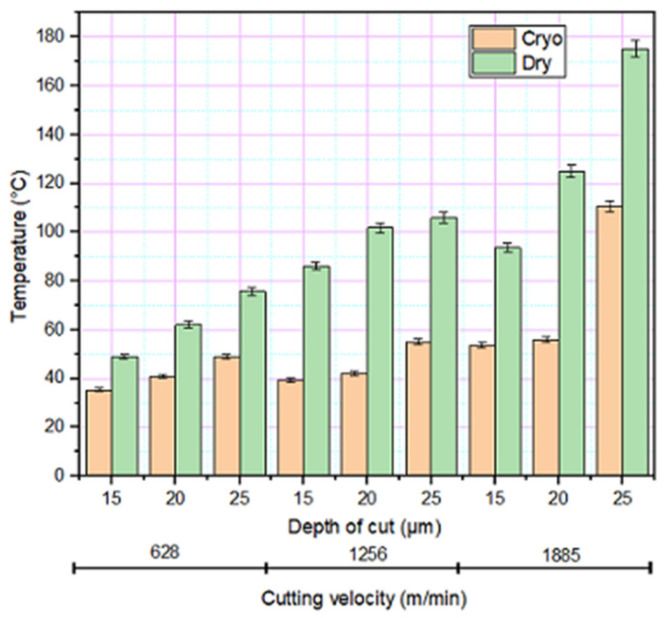
Variation in temperature under different grinding environments.

**Figure 4:**
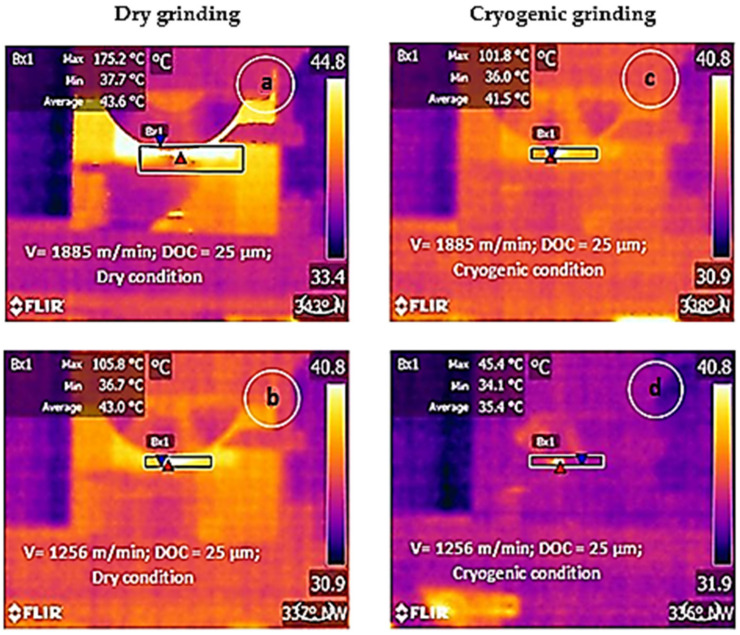
Thermal camera images captured under various grinding conditions (**a**,**b**) dry grinding; (**c**,**d**) cryogenic grinding.

In addition to grinding force, grinding zone temperature plays a significant role in determining the surface integrity of the material too. The factors such as velocity, feed, depth of cut, grit size and method of lubrication determine the amount of heat developed in the grinding zone [1]. The grinding zone temperature against the depth of cut with various cutting velocities is shown in Figure 3. With an increase in depth of cut and velocity, the grinding zone temperature increases due to friction [29]. During cryogenic grinding, the liquid nitrogen is sprayed on the wheel-work interface. The liquid nitrogen spray forms a thin layer of fluid cushion between grit and chip that effectively absorbs the heat generated during the grinding [37]. The thermal camera images during the experimental process are presented in Figure 4. The temperature developed during the cryogenic environment was about 35–45% less than dry grinding.

### 3.3. Surface Roughness

**Figure 5:**
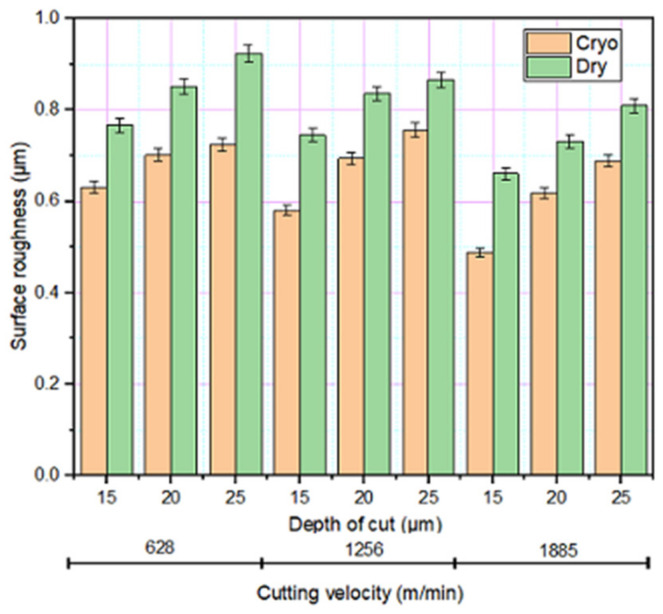
Variation in surface roughness under different grinding environments.

The surface roughness observed on the DMLS maraging steel with different cutting velocities, depth of cut and under different grinding conditions is illustrated in Figure 5. Average surface roughness (R_a_) was measured to assess the deviation under different conditions. As-built material had an initial surface roughness of 3.8–5.2 μm. With increased cutting velocity, a drop in surface roughness was observed. This is due to the reason that at higher cutting velocity and the related rise in grinding zone temperature, the ensuing bond dynamic is high as a result there will be a greater wheel-work interface which leads to better surface finish. While the depth of cut is increased, the greater penetration of the grinding wheel on to the workpiece increases the wear and wheel deformation that eventually resulted in increased roughness. In general, the sharpness of the grit plays a substantial role in reducing the roughness of the material; better the grit sharpness, better the surface roughness [27]. Increased surface roughness is observed at a higher depth of cut as the amount of undeformed chip thickness is more. The lowest surface roughness was observed under the cryogenic mode of grinding. The liquid nitrogen gas sprayed in between the tool-chip area, reduce the friction and thereby reducing the surface roughness. Additionally, under cryogenic grinding, the effective cooling of the workpiece has happened. This makes the material harder and reduced grinding zone temperature helps the grit to maintain its mechanical properties and retained sharpness throughout the grinding operation, all combined resulted in improved surface finish.

### 3.4. Microhardness

**Figure 6:**
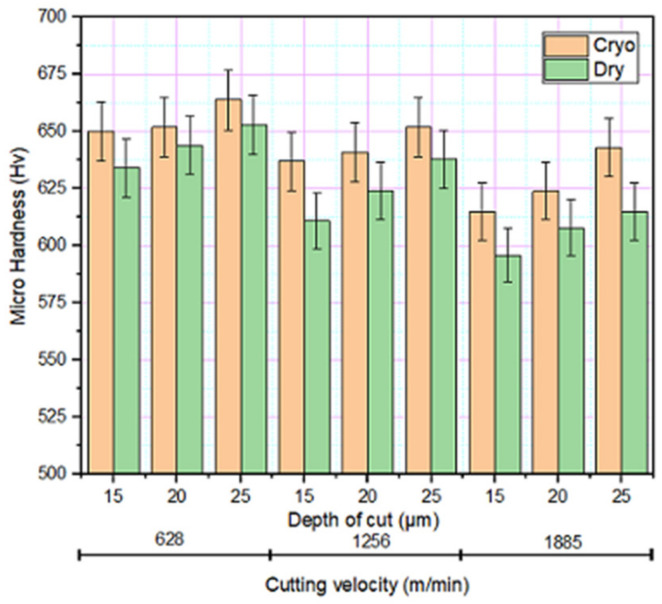
Variation of microhardness at different grinding environments.

The effect of various grinding parameters on the microhardness of the material is shown in Figure 6. The trend of decreasing microhardness with an increase in cutting velocity might be associated with the steady-state material removal which reduces the loading and thereby resulting in lower cutting force and eventually reduced surface hardness. The reverse trend is observed with increasing depth of cut. At a higher depth of cut, abrasive wear aggregates due to increased load for each abrasive particle which results in higher cutting force, thereby increasing the hardness. The temperature developed in cryogenic grinding due to the addition of liquid nitrogen is much lower compared to dry grinding. The cooling effect formed due to the spraying of LN_2_ makes the grinding area preserved, i.e., the area that is about to be ground will be frozen, which increases the hardness of the material. Besides, as the depth of cut and velocity surges, the temperature difference between the grinding area and coolant is high thereby increasing the hardness of the material [27].

### 3.5. Surface Residual Stress

**Figure 7:**
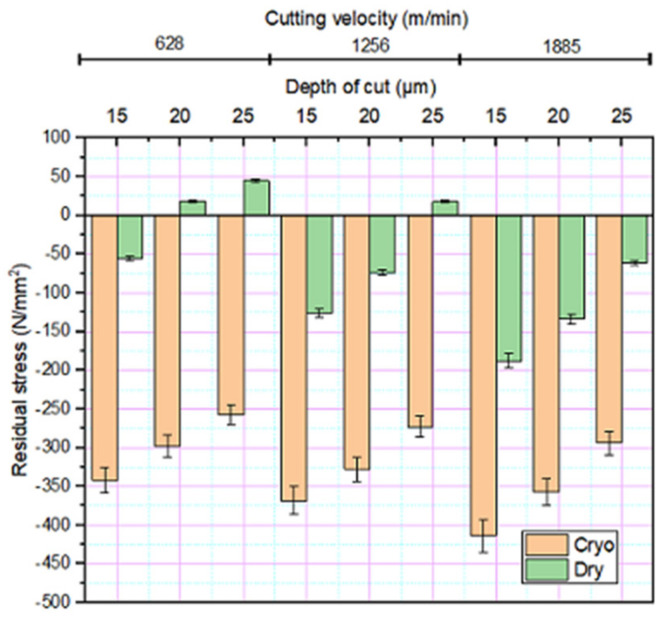
Variation in residual stress under different grinding environments.

The residual stresses imparted during printing were relieved through annealing. However, the residual stresses due to the grinding process cannot be ignored. The grinding process-induced residual stresses are resultant of complex thermo-mechanical and tribological process. Under low velocity and depth of cut, the plastic deformation induces predominantly compressive residual stresses. However, the temperature rise due to friction can lead to local expansion, which upon cooling induces tensile residual stresses. Apart from the grinding process parameters, the mechanical force and interface temperature during grinding is heavily influenced by the wear rate in the wheel/workpiece interface. In both dry and cryogenic grinding, compressive residual stress is found to increase with cutting velocity as inferred from Figure 7 indicating the influence of plastic deformation in the surface. However, as the grinding depth increases, the temperature rise in the interface induces tensile residual stresses. At the low velocity of 628 m/min, the compressive residual stress −50 MPa became tensile when the depth of cut increased from 15 to 20 μm. Under cryogenic grinding, the coolant reduces the effect of plastic deformation caused by thermal loading by governing the temperature developed in the grinding zone, thus making compressive residual stress more prominent [38].

### 3.6. Mechanical Testing

**Figure 8:**
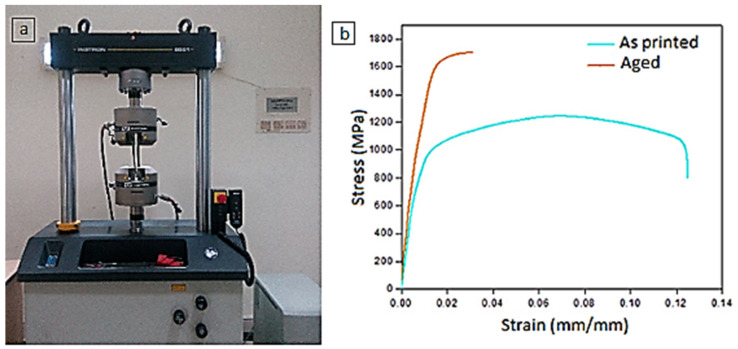
(**a**) Tensile testing set up (**b**) Stress–strain curve of maraging steel under as printed and aged conditions.

**Figure 9:**
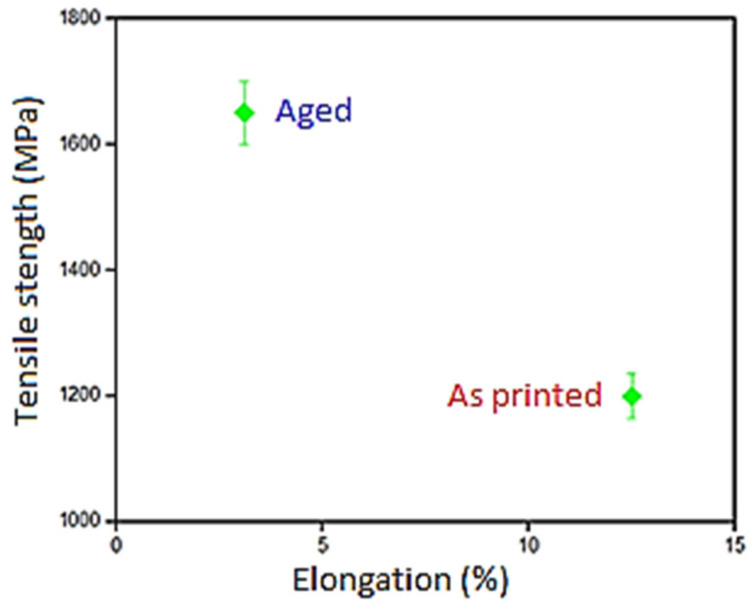
Variation of tensile strength and % elongation with aging treatment.

The tensile testing set up and the stress–strain curve obtained for maraging steel under as printed and aged conditions are presented in Figure 8a,b, respectively. The comparative evaluation of tensile strength and percentage elongation for these two samples were carried out. This is shown in Figure 9. The aged sample was found to be exhibiting about 36% higher tensile strength than the as-built sample. This is attributed to precipitation hardening [39]. During tensile testing, samples undergo deformation as a result of microcavities formed at imperfections and precipitates. Subsequently, these cavities coalesce and result in a fracture. The non-heat-treated samples were found to be broken after substantial plastic deformation favouring the ductile fracture, while the heat-treated samples not showing any plastic deformation and cause a considerable reduction in percentage elongation. This behaviour is coinciding with the test results reported in the literature [40,41]. The mechanical testing was performed on DMLS maraging steel specimens subjected to the post-processing under optimized conditions viz. cutting speed: 1885 m/min, DOC: 15 μm and environment: cryogenic. The optimization procedure carried out in this research work was elaborately detailed in Appendix A.

**Figure 10:**
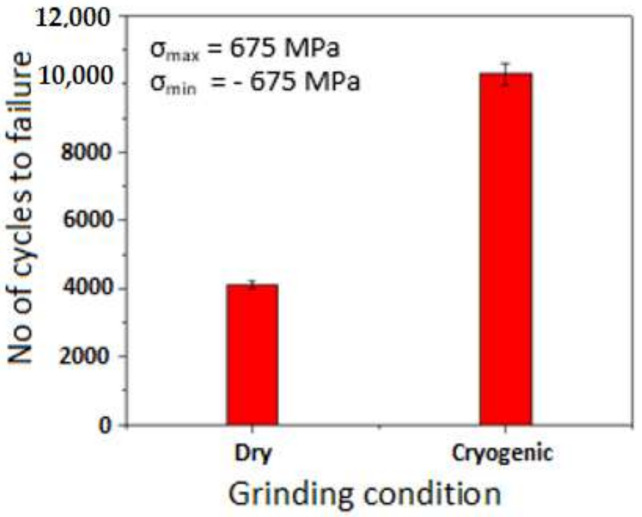
Fatigue test results for DMLS and aged maraging steel subjected to dry and cryogenic grinding.

In general, the fatigue life is influenced by the quality of the surface generated and the residual stress imparted in the material. The experimental results obtained from a fully reversed (R = −1) constant amplitude fatigue test with the optimum condition for the cryogenic and dry ground condition are shown in Figure 10. It has been established that the chemistry, heat treatment and cold working are considerably influencing the ultimate tensile strength and in turn the fatigue limit of the materials. Since most fatigue failures originate from the surface, the degree of surface finish plays a dominant role in deciding the fatigue behaviour of a material. Especially when the ultimate tensile strength and hardness of the materials are high as in the case of the present investigation, the fatigue limit is governed by the surface effects. A high degree of surface finish considerably increases the number of cycles required for the crack nucleation and thus the fatigue limit. Better surface finish achieved with cryogenic over dry grinding may be imputed to the rise in fatigue life of DMLS aged maraging steel.

The fatigue life of the material could be considerably improved by inducing compressive residual stresses [42]. A higher magnitude of compressive residual stresses was found to be induced under cryogenic grinding over the dry condition, which is also evident from Figure 7. In cryogenic grinding, the cooling occurs at a faster rate at the outer surface than the inner sider of the material. This faster thermal contraction at the outer induces tensile stress which is balanced by compressive stresses inside. A surface layer induced with compressive residual stress acts as an effective barrier to crack growth [43] and this stress is found to be effective in hampering the growth of microcracks started from the free surface of the material. Thus, more the compressive residual stress, more will be the number of alternating cycles required to cause the failure in the material. A high degree of surface finish and relatively higher magnitude of compressive residual stresses induced under cryogenic grinding are imputed to the increased fatigue life of DMLS maraging steel.

**Figure 11:**
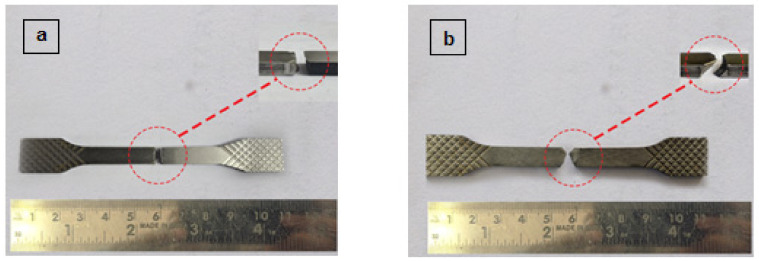
Sample after fatigue failure (**a**) Dry grinding (**b**) Cryogenic grinding.

**Figure 12:**
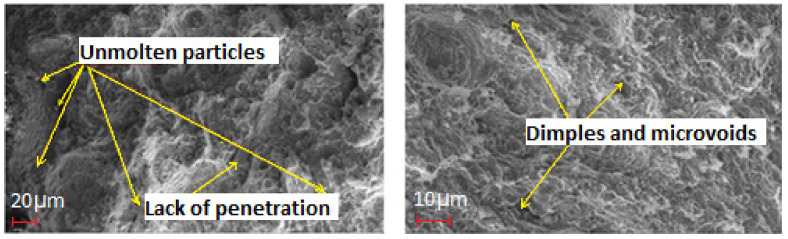
Fractographic analysis of samples.

The samples at the end of fatigue tests are shown in Figure 11. A kind of brittle fracture was observed during the fatigue test. This is associated with a reduced percentage of elongation caused by heat treatment. The examination of fractography reveals the presence of dimples, microvoids and unmolten particles. This is presented in Figure 12. Lack of fusion was observed which is caused as a result of improper deposition or melting rate. Generally, the area with a lack of fusion is non-symmetrical with sharp angles, which can act as a stress concentration area. While the specimen is loaded this stress concentration act as a crack initiator depending upon the orientation. Similarly, the microvoids propagation is causing a crack. These cracks will be smaller at the beginning and eventually emerge into a larger crack and ultimately causes the failure of the component upon loading [44].

## 4. Conclusions

A study on the influence of cryogenic grinding on the fatigue life of additively manufactured maraging steel is conducted. The different data, such as surface roughness, residual stress and forces generated during the grinding operation, are collected and examined. The outcomes of this research work are summarized and presented below.

1. Heat treatment of maraging steel at a temperature of 520 °C for 8 h significantly enhance the strength and hardness of the material. The precipitate formed during the heat treatment hinders the dislocation motion and thence improving the mechanical properties.
2. The additively manufactured maraging steel when further subjected to proper heat treatment exhibited an increase of about 36% in tensile strength.
3. Cryogenic mode of grinding extensively contributed to the reduced force and temperature in the grinding zone, thus reducing the surface roughness of the specimen. The surface roughness reduction considerably increases the number of cycles required to cause failure in the specimen, if the specimen is free from subsurface defects.
4. Cryogenic grinding avoids the plastic deformation due to thermal loading by dropping the temperature developed in the grinding zone, which will help to maintain the compressive residual produced as a result of plastic deformation caused due to mechanical loading and thus increases the number of cycles to cause a failure in the material samples.
5. The fractography analysis of fatigue samples revealed the presence of dimples, microvoids and unmolten particles. The fatigue life of additive manufactured components could be improved by about 170% time by adopting cryogenic grinding, which is revealed from the present work. The combined effect of meliorated compressive residual stress and better surface finish obtained through cryogenic grinding might be attributed to this improvement.
6. The composite desirability approach disclosed that grinding performed at a speed of 1885 m/min with a depth of cut of 15 μm under a cryogenic environment is producing favourable results in terms of surface roughness, forces generated and induced residual stress of DMLS maraging steels.

## Figures and Tables

**Table 1:** Material composition of MS1 powder.

Alloying Element	Ni	Mo	Co	Ti	Al	Cr	Mn	Si	Fe
Weight (%)	16–19	4.5–5.2	8.5–9.5	0.6–0.8	0.05–0.15	0–0.50	0–0.1	0–0.1	Bal.

**Table 2:** Grinding parameters.

Machine	NH 500 Surface Grinder
Grinding Wheel	CBN Metal Bond Wheel (Grade: B151)
Diameter of Wheel	200 mm
Width of Wheel	10 mm
Workpiece	Maraging Steel
Spindle Speed	628, 1256, 1884 m/min
Depth of Cut	15, 20, 25 μm
Environment	Dry & Cryogenic

**Figure A1:**
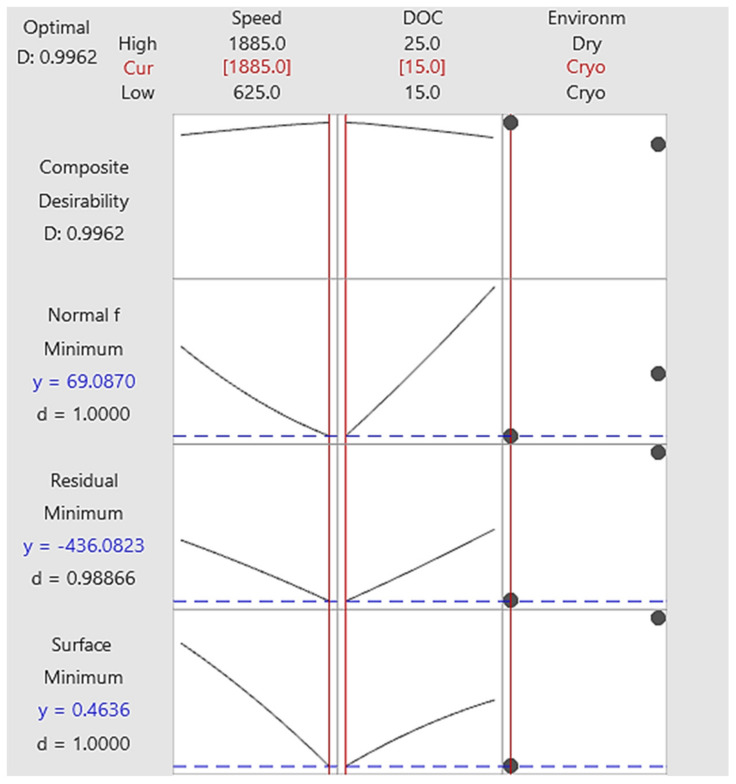
Multi-objective optimization plot for the grinding of DMLS maraging steel under different environments.

## Data Availability

Data can be made available upon request.

## Abbreviations

AM	Additive Manufacturing
CBN	Cubic Boron Nitride
EOS	Electro Optical System
TRS	Tensile Residual Stress
SLM	Selective Laser Melting
DML	Direct Metal Laser Sintering

## Appendix A

### Appendix A.1. Optimization Procedure

#### Appendix A.1.1. Regression Modelling

The regression model for the output responses during the grinding of Maraging steel under different grinding environments was carried out using MINITAB software (Version 19, Leandigit, Gurgaon, Haryana, India). Table A1 shows the factors and their levels used for optimization.

**Table A1:** Grinding parameters and levels.

Sl. No	Grinding Parameters	Levels		
		Low (−1)	Medium (0)	High (1)
1	Speed, S (m/min)	628	1256	1885
2	Depth of Cut, D (μm)	15	20	25
3	Environment Condition, C	Dry	-	Cryogenic

The regression model is considering the most significant terms that influence the output responses under different environments are presented below.

Under dry environment:

Surface roughness = 0.5409 − 0.000032 × S + 0.02010*D − 0.000227 ×D × D − 0.000002 × S × D (A1)

Normal force = 74 − 0.0637 × S + 7.7 × D + 0.000013 × S × S + 0.083 × D × D − 0.00087 × S × D (A2)

Residual stress = −105 − 0.1029 × S + 4.2 × D − 0.000008 × S × S + 0.097 × D × D + 0.00246 × S × D (A3)

Under cryogenic environment:

Surface roughness = 0.3872 − 0.000032 × S + 0.02010 × D − 0.000227 × D × D − 0.000002 × S × D (A4)

Normal force = 34 − 0.0637 × S + 7.7 × D + 0.000013 × S × S + 0.083 × D × D − 0.00087 × S × D (A5)

Residual stress = −369 − 0.1029 × S + 4.2 × D − 0.000008 × S × S + 0.097 × D × D + 0.00246 × S × D (A6)

The formulated regression model for surface roughness, normal force, and residual stress was found to have R_sq_ value of 99.41%, 90.86%, and 98.53%, respectively. The adequacy of the developed regression model is checked at a set speed of 1885 m/min and depth of cut of 15 μm. The percentage error was calculated between the regression model and experimental data and presented in Table A2. A maximum deviation of 12.88% was observed for the normal force between experimental data and regression model, while for other output responses, the deviations are comparatively lower, which confirms the adequacy of the model.

**Table A2:** Percentage deviation of regression model from experimental data.

Response	Experimental		Regression Model		Error (%)	
	Dry	Cryo	Dry	Cryo	Dry	Cryo
Normal Force (N)	110	80	108	69.6	1.8	12.88
Surface Roughness (μm)	0.624	0.467	0.674	0.520	7.4	10.19
Residual Stress (N/mm^2^)	−188	−414	−173	−437	7.2	5.2

#### Appendix A.1.2. Composite Desirability Approach for Multi-objective Optimization

The multi-objective optimization carried out in this work considered the grinding wheel speed and depth of cut as the input parameters while surface roughness, residual stress and normal force as the output parameters. The desired objective functions are (i) minimization of surface roughness, (ii) minimization of residual stress and (iii) minimization of the normal force. During optimization, higher weightage was given to surface roughness and residual stress over normal force as these parameters are playing a crucial role in deciding the fatigue life of additive manufactured components. Moreover, the intended aerospace application does not permit a compromise on the quality of finished maraging steel. The geometric mean of the desired value of output responses was calculated to find the composite desirability. The parameter which gave the highest composite desirability is considered as the optimum condition. The multi-objective optimization plot attained for DMLS maraging steel is shown in Figure A1.

From the plot, it is evident that the optimum parameters for the post-processing of DMLS maraging steel would be grinding performed at the cryogenic environment with a speed of 1885 m/min and depth of cut of 15 μm. For a reliable fatigue life comparison, the specimens were ground with these optimum grinding parameters under both environments.
